# Strengthening oil palm smallholder farmers’ resilience to future industrial challenges

**DOI:** 10.1038/s41598-024-62426-z

**Published:** 2024-05-27

**Authors:** Dienda Hendrawan, Daniel Chrisendo, Oliver Musshoff

**Affiliations:** 1 Department of Agricultural Economics and Rural Development, University of Göttingen, Göttingen, Germany; 2 Water and Development Research Group, Aalto University, Espoo, Finland

**Keywords:** Oil palm, Smallholder farmers, Resilience, Sustainable communities, Livelihood, Sustainability, Socioeconomic scenarios, Climate-change adaptation

## Abstract

Oil palm cultivation has improved living standards and alleviated the poverty of many smallholder farmers. However, challenges such as climate change, aging palms and negative sentiments in the major markets, threaten the wellbeing of and raise the question on smallholder farmers’ resilience, which remains poorly understood. Using primary data from Indonesia, the largest palm oil producer in the world, we measure and evaluate the resilience of oil palm smallholder farmers using the Sustainable Livelihoods Approach. Our results revealed five classes of smallholders with different levels of resilience: vulnerable, economically and socially constrained, low-skilled, semi-secure and adaptive smallholders. The farmers in the least resilient group are majorly older local farmers, who established oil palm plantations independently. Meanwhile, the most resilient group is dominated by smallholders who participated in the migration program, and in the past, received support from the government to start oil palm plantations. Our study highlights the heterogeneity of smallholders’ livelihood resilience and the need for inclusive and tailored interventions for the various classes of smallholder farmers to establish sustainable communities.

## Introduction

Oil palm, which produces the most widely-used and versatile vegetable oil in the world^1^, provides livelihood for up to 8 million people in Indonesia^2^, where it is mainly grown, with the smallholder farmers cultivating more than 40% of the oil palm plantation area^3^. Previous studies have shown that oil palm cultivation increases smallholder farmers' incomes by 14 to 25%^4^. These income gains then allow for higher living standards through improved education, nutrition, dietary quality, general living conditions, and asset ownership, which are essential factors for sustainable community formation^5^.

The palm oil industry faces upcoming challenges that might affect the smallholders’ wellbeing^6–10^. The temperature increase due to climate change accelerates the rate of soil water evaporation, thus resulting in drier soils, affecting soil nutrient absorption, disturbing the development of flowers, and lowering production^11^. An increase in temperature by 1–4 °C could reduce the oil palm yield by 10–40%, and in the next 70 years, the temperature in Southeast Asia is predicted to be extreme for oil palm^12^. Increasing threats from fungal diseases such as basal stem rot potentially reduce yields by 50–80%^13^. Smallholder-managed oil palm plantations have comparably lower yields compared to larger scale plantations^14^. Moreover, when the oil palms are around 25 years of age, they will start producing fewer fresh fruit bunches and need replanting^15,16^. Yet, many smallholders are struggling to fund replanting^14^. Although the increase in global population is likely to raise the demand for palm oil, concerns about the environmental sustainability of oil palm cultivation result in efforts to reduce the demand for palm oil, consumer boycotts and breakthroughs in electric vehicles^3,8,17,18^. The crude palm oil export from Indonesia to the European Union, a big market for Indonesian palm oil^19^, has decreased over the past decade^20^. Furthermore, labor shortages have arisen due to low wages and the growing appeal of alternative employment options, which can offer higher incomes^8,21^.

Though the palm oil industry undeniably plays a vital role in the country’s economy and smallholders^5^, it is still unclear how the smallholders could withstand, adapt and recover from ongoing and forthcoming challenges to maintain their living standards. A better understanding of various smallholders’ characteristics and resilience would help develop policies, mitigation, and adaptation measures and improve the sustainability of the palm oil industry and the community itself^22,23^, especially in places with limited income opportunities. Therefore, it is imperative to gain insights into their livelihoods and arrange the factors that impede their opportunities for sustenance^24^. Several studies have successfully developed typologies of Indonesian smallholder oil palm farmers, showing their different abilities to comply with sustainability standards^25,26^. However, empirical evidence concerning the resilience of various types of smallholders and their ability to mitigate and adapt to challenges are still poorly understood. Most socio-economic studies primarily focus on the positive effects of oil palm cultivation on livelihoods^4^ or specific institutional obstacles smallholders face^9^. Additionally, many oil palm studies focus solely on the environmental dimension rather than the sustainability of the smallholders’ livelihood and wellbeing in the community^27,28^. One study shows that oil palm smallholders are more resilient when facing fluctuating international oil prices^29^. However, the study only investigated economic resilience and did not observe the heterogeneous characteristics of oil palm smallholder farmers. To the best of our knowledge, no other study exists on the resilience of oil palm smallholder farmers to future palm oil industrial challenges.

This study comprehensively measured the livelihood resilience of Indonesian oil palm smallholders using the Sustainable Livelihoods Approach. This approach has often been used to capture vulnerability and measure livelihood resilience to enhance development programs^30^. It is constructed based on financial, human, natural, physical, and social capital aspects, which are regarded as building blocks for resilience^31,32^. Then, latent classes of smallholder farmers were identified based on their livelihood resilience levels to emphasize the importance of understanding its heterogeneity^33^. Finally, the most fitting adaptation and mitigation strategies for each identified latent class of smallholders were identified, and current relevant policies that may contribute to smallholder resilience were analyzed (e.g., sustainability certification, sustainable education). Interventions aimed at addressing tangible issues can be instrumental in disseminating knowledge and sustainable practices to foster sustainable communities^34^. To achieve our aim, we conducted a survey among 248 oil palm smallholder farmers in Jambi Province, Indonesia. Our local study from one of the oil palm expansion hotspots is relevant for the global community for two reasons. First, smallholders are important contributors to the global oil palm supply^35^. Second, palm oil is the most consumed and most versatile crop oil globally which receives the most attention inquiring about its sustainability measures^1^. We contribute insights on the social sustainability topic surrounding oil palm cultivation related to multiple Sustainable Development Goals.

## Theoretical framework for measuring resilience

The Sustainable Livelihoods Approach (SLA), developed by the British Department for International Development (DFID), emphasizes the interaction between five capital assets: financial, human, natural, physical and social (Table 1)^32^. The diverse capitals are building blocks for livelihood and resilience, in which one can withstand challenges or difficult situations and adapt and recover quickly to maintain wellbeing and continue functioning efficiently^36^. The diversity of livelihood capitals amplifies one’s capacity to respond to shocks and challenges, accumulate resources, give a broader range of options for decisions and actions, spread risk, and therefore bolster resilience^33^. Previous empirical research^33,37,38^ applied the SLA and acknowledged the multidimensional nature of this framework as a promising approach for capturing the assets and capabilities of humans. Our specific approach follows the study of Quandt^33^, who integrated subjective measures of livelihood resilience as indicators to measure each livelihood capital quantitatively. The quantitative indicators of each capital were adapted based on relevant literature and modified to fit the context of Indonesian oil palm smallholder farmers (Table 1).

**Table 1:** Five capital assets of SLA^32,39^ and measures of resilience^33,36^.

Livelihood asset	Definition	Quantitative indicator
Financial capital	Assets that can be diminished to build up other types of capital	Income from oil palm^a^
		Access to credits (yes or no)^33^
		Subsidy recipient (yes or no)
		Off-farm income^#^ (yes or no)
		Livestock unit^b^
Human capital	Capacity of an individual to work, including good health, knowledge, labor ability and skills	Number of household labor
		Household head education (years)
		Household head farming experience^c^
		Household head perceived health (healthy or not)
Natural capital	Stocks of natural resources that flow to produce ecosystem services as products that can be useful for livelihoods	Total farm area (hectares)
		Oil palm productivity^d^
		Amounts of other crops cultivated
		Perceived soil conditions (bad or good)
Physical capital	Human-made infrastructures, technology and tools that facilitate people's activities	Formal land certificate (yes or no)
		Motorbike ownership
		Car ownership
		Phone ownership^e^
		Living distance from capital city (kilometers)^f^
Social capital	Social relationships such as connections and networks, relations of trust and support, formal and informal groups, collective representation, mechanisms for participation in decision-making, leadership and influence	Living in an inti-plasma village (yes or no)
		Perceived political influence in the village (yes or no)
		Participation in farmer groups (yes or no)
		Participation in communities (yes or no)

^a^divided into 9 categories: 1 = IDR 0 to IDR 1.5 million, 2 = over IDR 1.5 million to IDR 3 million, 3 = over IDR 3 million to IDR 4.5 million, 4 = over IDR 4.5 million to IDR 6 million, 5 = over IDR 6 million to IDR 7.5 million, 6 = over IDR 7.5 million to IDR 9 million, 7 = over IDR 9 million to IDR 10.5 million, 8 = over IDR 10. 5 million to IDR 12 million, 9 = over IDR 12 million (fixed exchange rate: USD 1 = IDR 15,000). ^b^Livestock unit calculation indexes: cow = 1.0, sheep = 0.1, chicken = 0.014, duck = 0.01. ^c^divided into 6 categories: 1 = 0–5 years, 2 = 6–10 years, 3 = 11–15 years, 4 = 16–20 years, 5 = 21–25 years, 6 = more than 25 years. ^d^divided into 3 categories^41^: 0 = immature (0–5 years), 1 = less productive (between 5 and 7 years and over 25 years), 2 = productive (above 7 years until 25 years). ^e^divided into 3 categories: 0 = no phone, 1 = phone without internet, 2 = smartphone. ^f^distance by car, divided into 4 categories: 1 = 0 – 60 km, 2 = 61—120 km, 3 = 120 – 220 km, 4 = over 220 km. ^#^It is common for farmers to have more than one source of income in rural Indonesia, usually by working on other farmers’ or companies’ plantations or having a small business^39^.

The SLA itself has a weakness because it does not pay enough attention to inequalities of power^39^. In reality, diversity and variation of livelihood resilience exist among individuals engaged in small-scale agriculture that differentiate one smallholder from another^40^. In addition to the SLA, this study utilized latent class analysis to identify the unobserved heterogeneity and categorize the smallholders based on the unobservable groups within the population. By undertaking this approach, organizations and stakeholders are provided with the opportunity to identify and develop tailored interventions aimed at enhancing livelihood resilience among different groups of smallholders.

## Methodology

### Study area and data collection

The data used in this paper was collected in 2021 in Jambi Province, a major oil palm-producing province in Indonesia. With a population size of 217,000 oil palm smallholders^42^, Jambi is also a province with one of the highest shares of smallholders, which makes it a suitable place to conduct a study on smallholder farmers^43^. Given the study’s exploratory nature, the sample size was determined using a margin of error approach adequate to achieve a 95% confidence level with a 10% margin of error^44^. To accomplish this, a sample of 250 smallholders was selected from five oil palm high-producing regencies: Merangin, Muaro Jambi, Muaro Bungo, Batanghari and Tanjung Jabung Barat. Utilizing a stratified random sampling method, we randomly chose two districts from each regency. Subsequently, two villages were randomly selected from each district. Within each village, interviews were conducted with approximately 11 to 13 randomly chosen smallholder farmers. For the analysis, data from two respondents had to be dropped due to incomplete responses, resulting in a total sample size of 248 oil palm smallholder farmers. All respondents were at least 18 years old and owned less than 20 hectares of oil palm plantation. The interviews were conducted in the Indonesian language, face-to-face in one-on-one sessions by a team of local enumerators recruited, trained and supervised by the researchers. Respondents were told their participation was voluntary and information that may identify them would only be held on a secure server and not shared with third parties. Informed consent was obtained from all participants. All methods were carried out in accordance with relevant guidelines and regulations.

## Data analysis

The survey included questions about the sociodemographic characteristics of the respondents and other information relevant to livelihood assets important in oil palm cultivation (Table 1). Composite asset indexes for each respondent^33^ were created from the indicators in Table 1 to analyze the five capitals. The same rigorous composite asset index method was employed by the United Nations Development Programme to construct the Human Development Index^45^. Initially, the answers for each indicator question were converted to a scale of 0 to 1. A score of 0 was given to the response with the lowest value, while 1 was given to the response with the highest value. Concretely, for binary questions, such as participation in farmer groups, "no" received a score of 0, and "yes" received a score of 1. Regarding questions with continuous variable and multiple categories, responses were assigned values within the range of 0 to 1 by dividing the score of that respondent’s indicator by the sample average score of that indicator^33^. For example, if a respondent owns 5 hectares of plantation and the sample average is 10 hectares, then that respondent receives a score of 0.5 for the total farm area indicator. Therefore, the scores of one respondent’s indicators are relative to the scores of other respondents’ indicators. Next, the score results of all the indicators within a specific capital asset were averaged to form the composite asset indexes. For example, the results for all the indicators under financial capital were averaged to create an overall financial capital score for each respondent. Each indicator was assigned equal weight to simplify the analysis and reduce ambiguity. Lastly, the scores for each of the five capital composite indexes were averaged to create the overall resilience score for each respondent^33^. The literature suggested that higher scores of livelihood assets are related to higher livelihood resilience^33,46^. It is crucial to highlight that the indicator questions were not weighted. In reality, not all indicators hold equal importance, but assigning weights without substantial input from the research participant was not possible or appropriate^33^.

Latent class analysis was utilized to identify the unique profiles of smallholders. This statistical model is suitable when one assumes that a population consists of two or more underlying, latent subgroups defined by the intersection of various individual characteristics^47^. In this analysis, a mixture model is applied, postulating the presence of an underlying, unobserved categorical variable. This variable categorizes the population into mutually exclusive and exhaustive latent classes based on a set of observed items, determining the class membership of individuals^47^.

The latent class model can be expressed as

$$p{i}_{1},{i}_{2},\dots {i}_{n}= {\sum }_{c=1}^{C}{p}_{c}{\prod }_{n}^{5}{p}_{{i}_{n},c}^{n}$$

where *C* is the number of latent classes, and n *(n* = *1,2,…5)* represents the five livelihood assets (financial, human, natural, physical and social capial) , and ${p}_{c}$ is the probability of recruitment or respondent *i* to class *C* or unconditional probabilities that should sum to one. ${p}_{{i}_{n},c}^{n}$ are the probability of response conditional on membership in class *C*.

Latent class analysis for 4-class, 5-class and 6-class models were conducted. Based on the Bayesian Information Criterion (BIC), which is an estimator that estimates the quality of a model relative to other models^48^, we selected the best fitting model. The BIC values suggested a model with five classes (C = 5) since the BIC value for a 5-class model (− 824.11) is lower than the BIC value for a 4-class model (− 822.67) and lower than the BIC values for a 6-class model (− 806.75) (Table S1 in the Supplementary Information). Each respondent was assigned to the class in which their membership probability was the largest. Following the identification of the latent classes, one-way ANOVAs with post hoc Tukey’s tests^49^ were used to determine the differences in age, self-assessed risk attitude^50^, and average plantation age between the five identified latent classes.

## Results

### Descriptive results

The household characteristics of the respondents are presented in Table S2 in the Supplementary Information. All of the 248 respondents were oil palm smallholders with productive plantations, where 51% of them live in villages that initially got support to establish oil palm plantation, or so called inti-plasma villages^51^. The respondents were either household heads or household decision-makers. About 16% were female and 39% had a migration background. The respondents were, on average, 45 years old and had received nine years of formal education. The respondents, on average, scored 5.29 on a self-assessed risk attitude^50^ from the scale of 0 (not willing at all to take risks) – 10 (very willing to take risks). About 25% were new oil palm smallholders with less than five years of experience, while about 12% had over 25 years of experience in oil palm cultivation. A typical household had four working and financially productive adults. The average oil palm land holding was 3.41 hectares per household and 73% farmers have formal land certificates. The average oil palm productivity of the respondents’ farms was between the category of less productive to productive. The average plantation age is 12.25 years.

### Underpinning factors of vulnerability and improvement priorities for smallholders with diverse livelihood resilience

**Figure 1:**
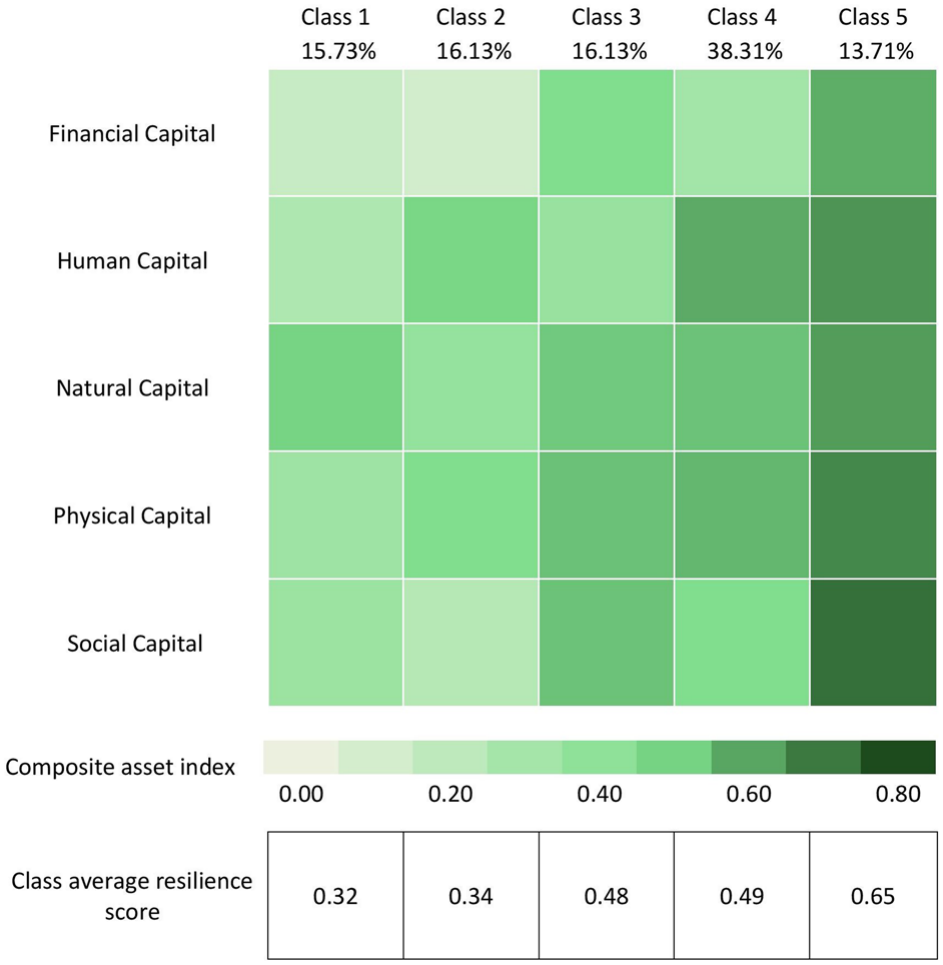
Typologies of smallholders based on latent class analysis (n = 248).

**Figure 2:**
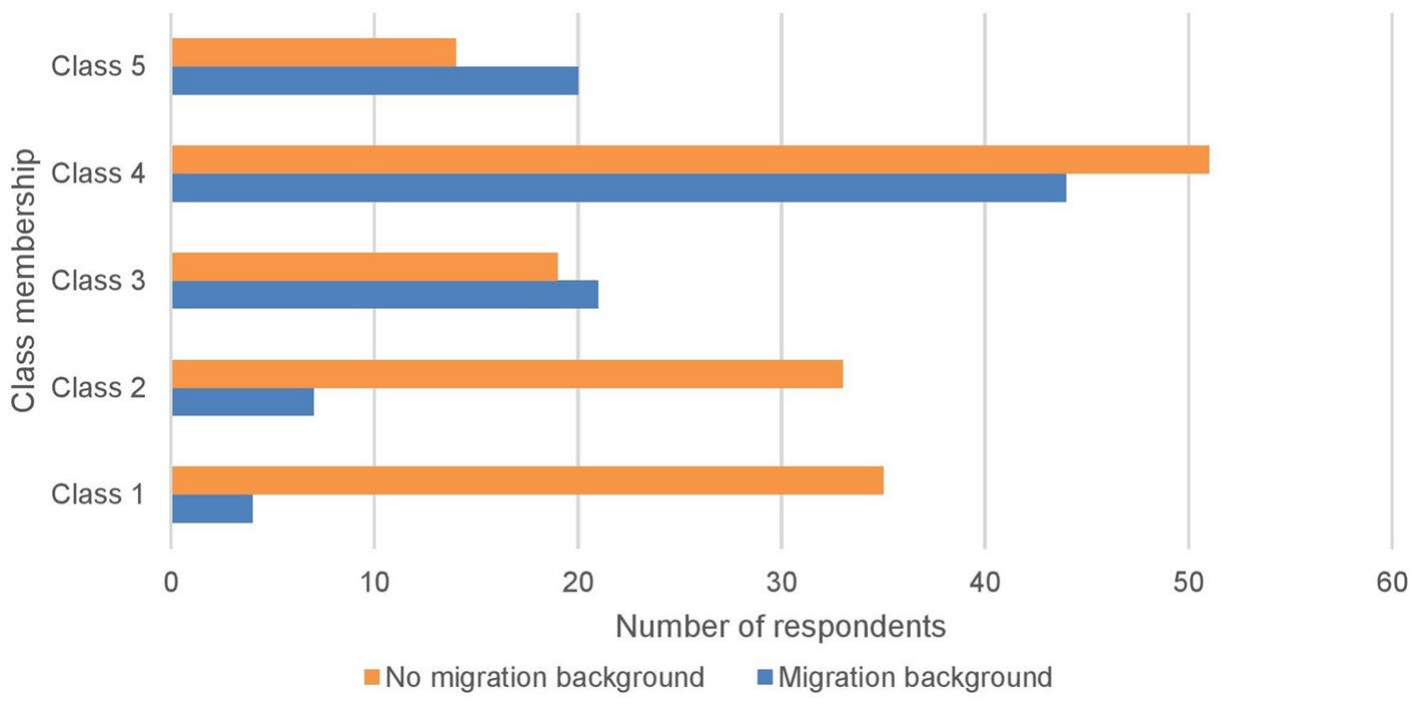
Smallholders’ class membership based on the migration background (n = 248).

Latent class analysis revealed the unobserved heterogeneity within the oil palm smallholder community and divided the smallholders into five classes based on their livelihood resilience (Fig. 1). The interpretation and labeling of each subgroup were based on the information derived from their respective parameter estimates, as demonstrated by Lanza^47^. In our study, the parameter estimates are the composite capital index scores (Table S3 in the Supplementary Information). Among the smallholders, 15.73% (n = 39) fall into Class 1 “vulnerable smallholders”, 16.13% (n = 40) into Class 2 “economically and socially constrained smallholders”, 16.13% (n = 40) into Class 3 “low-skilled smallholders”, 38.31% (n = 95) into Class 4 “semi-secure smallholders”, and 13.71% (n = 34) into Class 5 “adaptive smallholders”. The first two classes are the least resilient. Most of the smallholders in these classes are smallholders who do not have a migration background or are so-called local farmers (Fig. 2) and mainly established oil palm plantations independently. Meanwhile, Class 5 is the most resilient, consisting mostly of migrant smallholders (Fig. 2) who participated in the Indonesian government’s transmigration program, and in the past, received extensive support from the government to start oil palm plantations, such as agricultural inputs and access to the market.

Our results show that smallholders exhibit considerable heterogeneity in livelihood resilience, meaning that the suitability and receptivity to adopting proposed adaptation and mitigation strategies to improve livelihood resilience and maintain wellbeing may vary. Key considerations include whether smallholders can understand, possess the capacity to implement the intervention, utilize the benefits optimally, or require assistance or another form of intervention instead.

Class 1: vulnerable smallholders

Class 1 smallholders, labeled as vulnerable, have low financial and human capital, as well as limited physical and social capital. The members of this class, the least resilient among smallholders, bear the full weight of challenges with the lowest average overall resilience score. The low financial and human capital reflects sparse access to financial resources like savings, credit, or assets and lower levels of formal education, skills, or health status to perform well in agricultural activities. This, in turn, makes it difficult for them to cope with and adapt to numerous shocks and challenges they face. Furthermore, their limited physical and social capital worsens their situation as they may lack sufficient of physical assets and struggle to rely on their social networks when faced with adversity.

To truly enhance the resilience of vulnerable smallholders, it is imperative that policy interventions adopt a comprehensive approach. This approach should first and foremost focus on covering and strengthening the financial and human capacity of these smallholders. This could be done through direct financing, such as providing direct financial aid or subsidies, and capacity-building interventions, such as farmer extension support for farming management practices. While knowledge enhancement and increased motivation are important, they alone may not be sufficient for smallholders to achieve resilience. Therefore, a combination of financial support and capacity building, facilitated through policy interventions, is crucial to enhance their ability to withstand and recover from challenges^52,53^.

Class 2: economically and socially constrained smallholders

Class 2 smallholders are labeled as economically and socially constrained due to their low social capital and limited financial resources. They may struggle to meet basic needs and invest in their oil palm plantations. The lack of robust social networks and support systems further isolates them, potentially requiring them to overcome challenges independently. However, their higher human capital compared to Class 1 and Class 3 smallholders suggests they may be better equipped to rely on their inner capacity to cope and adapt to shocks and challenges when social support is lacking.

To address challenges faced by these smallholders, policy interventions should focus on strengthening their lack of financial and social capital respectively. Direct financing, such as providing financial aid or subsidies^54,55^, can be helpful, along with community-based initiative that enhance social capital. Given their isolation, efforts to build social capital should be prioritized. Establishing and supporting farmer groups and cooperatives can provide access to resources and networks that enhance resilience. Community-based approaches, such as group workshops, knowledge-sharing platforms, and collective action can further support these smallholders in improving their resilience to various challenges^56^.

Class 3: low-skilled smallholders

Class 3 farmers, labeled as low-skilled smallholders, face a high probability of the members having low human capital. Compared to Class 2 smallholders, they have more financial, natural, physical and social capital. However, their limited education, skills and human resources, require capacity-building interventions such as educational and training programs that can improve their human capital for enhancing their agricultural productivity and livelihoods. Compared to Class 1 and Class 2 smallholders, the moderately higher possessions of other capitals provide this class with more options for safety nets when facing shocks and challenges. For example, their stronger social network and external support system, reflected by the social capital score, can provide information, resources and new opportunities. Similarly, the higher financial capital score reflects the better availability of cash buffer when there are shocks such as harvest failures or price fluctuations.

Policy interventions for low-skilled smallholders could focus on capacity building initiatives, such as providing training programs on farming techniques, crop management and sustainable agricultural practices^57^. Despite their adequate social and financial capital, which can be utilized to substitute for personal capacity by leveraging their support systems and financial resources for farm management tasks, there is still a need for improvement in their human capital. Given their strong social networks, efforts to enhance human capital could be managed through collective action, leveraging their existing support systems to facilitate skill development and knowledge enhancement.

Class 4: semi-secure smallholders

Class 4 smallholders are labeled as semi-secure smallholders due to the high probability of the members having strong possessions of human, natural, physical and social capital but lower financial capital compared to other classes. These smallholders might still face financial limitations for sustaining their daily lives and investing in their farms, although not as critical as Class 1 and Class 2 smallholders. Compared to Class 3 and Class 5 smallholders, the social capital score of this class is lower, indicating the need for more support from external or social networks. Based on the livelihood resilience score, they are more protected against certain risks or threats compared to Class 1, 2 and 3, but not to the extent of complete security. They may still be vulnerable to certain shocks and challenges, especially direct financial shocks such as price volatility, which can impact their income and ability to invest in their farms, leading to a cycle of poverty and insecurity.

Policy interventions for semi-secure smallholders could focus on supporting smallholders through community-based entrepreneurship programs to expand financing options and enhance their financial security^58^. These programs, for instance, could offer training in business skills and access to financial resources, empowering smallholders to initiate or expand their agricultural businesses. Moreover, efforts to bolster social capital could involve facilitating the formation and activities of farmer cooperatives or other community-based organizations. These initiatives would not only promise to improve the financial security of semi-secure smallholders but also to strengthen their social networks, thereby enhancing their overall resilience.

Class 5: adaptive smallholders

Class 5 smallholders, distinguished as adaptive, demonstrate the highest overall resilience score among the five classes. Their comprehensive capabilities across all five capitals underscore their exceptional potential to adapt and sustain their livelihoods in the face of challenges, stresses and shocks. This class, best-equipped, can adjust and respond effectively to various shocks and challenges, with a better capacity to innovate, learn and adopt new strategies in response to factors such as climate variability, market prices or resource availability.

Class 5 smallholders, in their proactive approach to bolster resilience, could consider adopting oil palm sustainability certification programs such as Roundtable on Sustainable Palm Oil (RSPO) to enhance market access and profitability. They could also prepare their finances and technical farming management skills for replanting, minimizing income loss and adapting to changing climatic conditions. Furthermore, implementing Good Agricultural Practices (GAP) or sustainable farming practices for long-term viability is a key strategy^59^. Leveraging their strong foundation across all five capitals, these adaptive smallholders are not just surviving but also thriving in challenging agricultural landscapes.

## Discussion

Smallholders in the oil palm industry exhibit a spectrum of resilience, which is crucial to navigate through risks, threats and shocks. This diversity underscores the need to recognize and understand the varying levels of resilience among smallholders. Doing so allows us to identify the more vulnerable groups who may require additional attention and assistance to enhance their adaptive capacity. As discussed earlier, each class of smallholders has distinct priorities for enhancing their livelihood capitals. The five livelihood capitals play varying roles in bolstering smallholders' resilience when confronted with shocks or challenges (Table 2). Strategically addressing the dimensions of these capitals through tailored interventions that cater for the unique needs of each class of smallholder, ensures the effectiveness of efforts to build resilience incrementally.

**Table 2:** Importance of the five livelihood capitals.

Capital	Importance
Financial	Financial resource for farm management practices^14^
	Buffer for income loss^61^
	Recovery costs^62^
	Allows for diversification of income sources^63^
Human	Physical ability to work (manpower)^64^
	Problem solving and decision making^64^
	Knowledge, adaptation and innovation^65^
	Crisis management^66^
Natural	Provision of ecosystem services^67^
	Reduce disaster risk^68^
	Crop growth and productivity^69^
	Income diversification^28^
Social	Information flow and knowledge exchange^70,71^
	Resource sharing and collaboration^72^
	Practical and financial support system^73^
	Increased adaptive capacity^74^
Physical	Communication system^75^
	Transportation networks^76,77^
	Emergency response assets^78^

Citations indicate scientific publications stating the importance of the related capital.

By recognizing the different levels of smallholders’ resilience, interventions can be designed to be more targeted and inclusive to account for the different capabilities of varying smallholder classes. For example, smallholders are facing the declining productivity of aging oil palm and would need to replant^14^. However, smallholders with limited financial capital have limited financial resource for farm management practices, and might not be able to afford replanting independently. The Indonesian government has initiated a subsidy program to assist smallholders to fund replanting^60^. While financial-capital-enhancing support is available, not all smallholders in Class 1 and Class 2 can meet the requirements to receive the support. Most of these smallholders are local farmers who might not have official land certificates required to participate in the replanting subsidy program and also many other farming management support subsidy programs. Meanwhile, smallholders who were part of the government transmigration program in the 1980s, were given around 2 ha of land with official land certificates to cultivate oil palm^42,51^. These certificates can also be used as collateral for bank loans, not only for funding replanting, but also for funding other farming management practices and farm improvements. The lack of formal land certificates contributes to the low physical capacity, and therefore lower resilience levels of Class 1 and Class 2 smallholders. Class 1 and Class 2 smallholders currently have the youngest average plantation age (Table S4 in the supplementary information), which may also indicate that they are not only local, but also independent smallholders who more recently switched to oil palm.

The heterogeneity of smallholders’ resilience levels emphasizes that not all interventions are universally effective. To enhance the environmental sustainability of the global palm oil production and provide opportunities for smallholders to receive better incomes, stakeholders have developed RSPO certification. Regardless of the good intentions, obtaining RSPO certification can be costly and time-consuming, particularly for smallholders and producers in developing countries^59^. Smallholders with limited social and financial capital will counter difficulties in conducting the pre-assessments of plantations, meeting the registration requirements and funding the certification. For instance, to adopt RSPO, smallholders must register in groups. Those without strong social networks to gather essential information and form groups will find it challenging^54^. Hence, the likelihood of Class 1, Class 2, Class 3 and Class 4 smallholders to adopt RSPO certification is lower compared to Class 5 smallholders, who possess higher levels of the five capitals. In addition, Class 1 smallholders are, on average, older (Table S4 in the supplementary information). Older farmers may be less aware of new technology and programs such as RSPO. Although RSPO certification offers benefits for sustainable palm oil production, its accessibility challenges underscore the necessity for inclusive measures to support smallholders in adopting sustainable practices^54^.

The apparent differences in resilience between farmers who received and did not receive support underline the importance of inclusive and tailored interventions in strengthening oil palm smallholders’ resilience. Resilience can be developed over time through learning and adaptation^79^. Sustainable education has surfaced as one of the fundamental strategies in shaping a more sustainable future civil society^34^. For farmers, sustainable education interventions must be relevant and should be practical, enabling them to acquire the knowledge and skills necessary to adapt and cope with risks, threats and shocks. Effective sustainable education takes various forms including technical training for better farming management practices, farm resources management, knowledge sharing, initiation of value-added opportunities, as well as community engagement, which are important for strengthening resilience to future industrial challenges and building sustainable smallholder communities. Among the numerous approaches for sustainable education, farmer field schools^80^ present an excellent option for inclusive sustainable education among farmers. These schools offer hands-on learning experiences directly in the field, allowing farmers to observe, experiment and learn from each other under the guidance of trained facilitators. In addition to enhancing human capital primarily, this approach promotes peer-to-peer knowledge exchange and cultivates social trust to improve social capital^56^. The quality of social interactions and trust contributes to the development of sustainable communities, encourages innovation and supports the growth of local economies^81,82^. With greater social trust, farmer communities can collaborate more closely with stakeholders or higher educational institutions (HEIs) to develop research collaborations, outreach programs and workshops focusing on educational activities to build resilience step-by-step.

## Conclusion

With rising global industrial challenges on oil palm cultivation, the resilience of oil palm smallholder farmers is worth investigating to understand whether their livelihood is sustainable. In this study, the farmers livelihood resilience was measured and five different classes of smallholders based on their livelihood resilience levels were identified: (i) vulnerable, (ii) economically and socially constrained, (iii) low-skilled, (iv) semi-secure and (v) adaptive smallholders. We show that the least resilient group are older local farmers, who mainly established oil palm plantations independently. Meanwhile, the most resilient group is dominated by smallholders who participated in the migration program and, in the past, received support from the government to start oil palm plantations. Despite the same core challenges that all classes of smallholders face, navigating risks, threats and shocks through inclusive tailored policy for oil palm smallholders with varying levels of resilience is crucial to ensuring the sustainability and stability of every class of smallholders. Policies and interventions should be formulated to address distinct adaptation requirements and tailored to accommodate smallholders’ unique adaptation needs. More stakeholders should take part in supporting smallholders as they are the most vulnerable group in the oil palm value chain because bolstering the resilience of smallholders also supports the sustainability of the palm oil industry. With better resilience, smallholders have a greater chance to avoid the poverty trap, improve their management practices to improve yield and contribute to the environmental sustainability of the palm oil industry. Tailored policy guidance, and non-formal education interventions such as farmer field schools can help mitigate negative environmental effects, enhance shock preparedness, and promote resilience among smallholders, thereby safeguarding their livelihoods and the broader socio-economic landscape. A robust livelihood resilience of smallholder farmers is crucial to achieving sustainable communities in the oil palm industry.

### Supplementary Information

Supplementary Information.

## Data Availability

Anonymized data are available on reasonable request from the corresponding author.
