# Supplementary material for: Strengthening oil palm smallholder farmers’ resilience to future industrial challenges
Source: Sci Rep. 2024 May 27;14:12105. doi: 10.1038/s41598-024-62426-z (PMC11130153; doi:10.1038/s41598-024-62426-z)
Supplement: Supplementary file 1 — Supplementary Information. [file 41598_2024_62426_MOESM1_ESM.pdf]

Title: Strengthening oil palm smallholder farmers‘ resilience to future industrial challenges

Authors

Dienda Hendrawan (corresponding author)

Department of Agricultural Economics and Rural Development, University of Göttingen, Germany, email: dienda.hendrawan@uni-goettingen.de, ORCID: 0000-0002-6149-728X

Daniel Chrisendo

Water and Development Research Group, Aalto University, Finland, ORCID: 0000-0002-4941-8606

Oliver Musshoff

Department of Agricultural Economics and Rural Development, University of Göttingen, Germany, ORCID: 0000-0002-3746-623X

Supplementary Information

Table S1. Model selection based on Bayesian Information Criterion (n = 248)

Model	ll	df	BIC
4-class	488.52	28	-822.67
5-class	505.78	34	-824.12
6-class	513.64	40	-806.75

Table S2. Household characteristics of smallholder oil palm farmers based on different capitals (n=248)

Livelihood asset	Variable	Mean	SD	Min	Med	Max
Financial capital	Income from oil palm[a]	3.39	2.37	1	3	9
	Access to credits (yes=1, no=0)	0.68	0.47	0	1	1
	Subsidy recipient (yes=1, no=0)	0.29	0.45	0	0	1
	Income outside oil palm (yes=1, no=0)	0.37	0.48	0	0	1
	Livestock unit (LU)[b]	0.71	1.77	0	0.14	18.56
Human capital	Number of household labor	1.65	0.83	1	1	5
	Household head years of education	9.02	3.83	0	9	19
	Household head farming experience[c]	2.97	1.66	1	3	6
	Perceived health of household head (healthy=1, not=0)	0.68	0.47	0	1	1
Natural capital	Total farm area (hectares)	3.40	2.99	0.25	2.3	18
	Oil palm productivity[d]	0.68	0.41	0	1	1
	Amounts of other crops cultivated	4.13	3.14	0	4	15
	Perceived soil conditions (good=1, bad=0)	0.92	0.27	0	1	1
Physical capital	Formal land certificate (yes=1, no=0)	0.73	0.44	0	1	1
	Motorbike ownership (n)	2.27	1.14	0	2	6
	Car ownership (n)	0.40	0.72	0	0	4
	Phone ownership[e]	1.55	0.70	0	2	2
	Living distance from capital city[f]	2.51	1.13	1	3	4
Social capital	Living in an inti-plasma village (yes=1, no=0)	0.51	0.50	0	1	1
	Perceived political influence in the village (yes=1, no=0)	0.19	0.39	0	0	1
	Participation in farmer groups (yes=1, no=0)	0.36	0.48	0	0	1
	Participation in communities (yes=1, no=0)	0.76	0.43	0	1	1
Other variables	Age of respondent (years)	45.29	10.89	21	45	76
	Plantation age (years)	12.25	8.02	0	10	35
	Risk attitude	5.29	3.23	0	5	10
	Gender (female=1, male=0)	0.16	0.36	0	0	1
	Migration background (yes=1, no=0)	0.39	0.49	0	0	1

Notes: [a]divided into 9 categories: 1 = IDR 0 to IDR 1.5 million, 2 = over IDR 1.5 million to IDR 3 million, 3 = over IDR 3 million to IDR 4.5 million, 4 = over IDR 4.5 million to IDR 6 million, 5 = over IDR 6 million to IDR 7.5 million, 6 = over IDR 7.5 million to IDR 9 million, 7 = over IDR 9 million to IDR 10.5 million, 8 = over IDR 10. 5 million to IDR 12 million, 9 = over IDR 12 million (fixed exchange rate: 1 USD = IDR 15,000) [b]Livestock unit calculation indexes : cow = 1.0, sheep = 0.1, chicken = 0.014, duck = 0.01. [c]divided into 6 categories: 1 = 0-5 years, 2 = 6-10 years, 3 = 11-15 years, 4 = 16-20 years, 5 = 21-25 years, 6 = more than 25 years. [d]divided into 3 categories[40]: 0 = immature (0-5 years), 1 = less productive (between 5-7 years and over 25 years), 2 = productive (above 7 years until 25 years). [e]divided into 3 categories: 0 = no phone, 1 = phone without internet, 2 = smartphone. [f]distance by car, divided into 4 categories: 1 = 0 – 60 km , 2 = 61 - 120 km, 3 = 120 – 220 km, 4= over 220 km.

Table S3. Parameter estimates (n=248)

**Class**	**Livelihood asset**	**Margin**	**Std. error**	**z**	**P>|z|**	**95% conf. interval**	
Class 1	Financial capital	0.16	0.03	6.41	0.00	0.11	0.21
	Human capital	0.27	0.02	16.09	0.00	0.24	0.30
	Physical Capital	0.34	0.03	11.26	0.00	0.28	0.40
	Social Capital	0.35	0.04	8.66	0.00	0.27	0.43
	Natural Capital	0.50	0.03	19.19	0.00	0.45	0.55
Class 2	Financial capital	0.13	0.03	4.53	0.00	0.08	0.19
	Human capital	0.49	0.02	24.57	0.00	0.45	0.53
	Physical Capital	0.47	0.03	16.21	0.00	0.41	0.53
	Social Capital	0.25	0.05	5.39	0.00	0.16	0.33
	Natural Capital	0.39	0.03	11.80	0.00	0.33	0.46
Class 3	Financial capital	0.44	0.03	14.69	0.00	0.39	0.50
	Human capital	0.37	0.02	16.26	0.00	0.33	0.42
	Physical Capital	0.54	0.03	19.85	0.00	0.48	0.59
	Social Capital	0.53	0.04	12.10	0.00	0.45	0.62
	Natural Capital	0.51	0.03	19.22	0.00	0.46	0.56
Class 4	Financial capital	0.31	0.02	12.84	0.00	0.27	0.36
	Human capital	0.59	0.02	39.31	0.00	0.56	0.62
	Physical Capital	0.57	0.02	30.62	0.00	0.53	0.60
	Social Capital	0.46	0.03	13.91	0.00	0.40	0.53
	Natural Capital	0.54	0.02	30.82	0.00	0.51	0.58
Class 5	Financial capital	0.57	0.03	18.24	0.00	0.51	0.63
	Human capital	0.64	0.02	36.60	0.00	0.60	0.67
	Physical Capital	0.66	0.03	24.63	0.00	0.61	0.71
	Social Capital	0.70	0.05	14.86	0.00	0.61	0.79
	Natural Capital	0.61	0.03	24.01	0.00	0.56	0.66

Notes: Class 1 = 15.73% (n=39), Class 2 = 16.13% (n=40), Class 3 = 16.13% (n=40), Class 4 = 38.31% (n=95), Class 2 = 13.71% (n=34)

Table S4. Cross-class comparisons on age, risk attitude, and plantation age.

**Variable**	**Class 1**	**Class 2**	**Class 3**	**Class 4**	**Class 5**	**F statistic**	***p*-value**	**Significant pairwise post hoc tests[a]**
Age	51.92 (9.68)	42.55 (11.00)	49.10 (9.51)	42.83 (11.02)	45.29 (10.89)	7.69	0.00***	Class 1 > Class 2<br>Class 1 > Class 4<br>Class 1 > Class 5<br>Class 3 > Class 2<br>Class 3 > Class 4
Risk attitude	4.41 (3.44)	4.8 (3.65)	5.18 (3.14)	5.53 (3.06)	6.38 (2.84)	2.09	0.08	n/a
Plantation age	10.95 (6.62)	6.90 (5.97)	13.29 (7.67)	13.30 (8.74)	15.94 (6.79)	7.88	0.00***	Class 3 > Class 2<br>Class 4 > Class 2<br>Class 5 > Class 1<br>Class 5 > Class 2

Notes: Data report means, standard deviation in parentheses. ***Statistically significant differences between classes (5% level). [a]Non-significant pairwise post hoc Tukey's test are not reported.
